# Racial Differences in Genetic and Environmental Risk to Preterm Birth

**DOI:** 10.1371/journal.pone.0012391

**Published:** 2010-08-25

**Authors:** Timothy P. York, Jerome F. Strauss, Michael C. Neale, Lindon J. Eaves

**Affiliations:** 1 Virginia Institute for Psychiatric and Behavioral Genetics, Virginia Commonwealth University School of Medicine, Richmond, Virginia, United States of America; 2 Department of Human and Molecular Genetics, Virginia Commonwealth University School of Medicine, Richmond, Virginia, United States of America; 3 Department of Obstetrics and Gynecology, Virginia Commonwealth University School of Medicine, Richmond, Virginia, United States of America; 4 Department of Psychiatry, Virginia Commonwealth University School of Medicine, Richmond, Virginia, United States of America; Universidad Europea de Madrid, Spain

## Abstract

Preterm birth is more prevalent in African Americans than European Americans and contributes to 3.4 times more African American infant deaths. Models of social inequity do not appreciably account for this marked disparity and molecular genetic studies have yet to characterize whether allelic differences that exist between races contribute to this gap. In this study, biometrical genetic models are applied to a large mixed-race sample consisting of 733,339 births to measure the extent that heritable factors and environmental exposures predict the timing of birth and explain differences between racial groups. Although we expected significant differences in mean gestational age between racial groups, we did not anticipate the variance of gestational age in African Americans (σ^2^ = 7.097) to be nearly twice that of European Americans (σ^2^ = 3.764). Our results show that this difference in the variance of gestational age can largely be attributed to environmental sources; which were 3.1 times greater in African Americans. Specifically, environmental factors that change between pregnancies, versus exposures that influence all pregnancies within a family, are largely responsible for the increased reproductive heterogeneity observed in African American mothers. Although the contribution of both fetal and maternal genetic factors differed between race categories, genetic studies may best be directed to understanding the differences in the socio-cultural sources of this heterogeneity, and their possible interaction with genetic differences within and between races. This study provides a comprehensive description of the relative genetic and environmental contributions to racial differences in gestational age.

## Introduction

Preterm birth, defined as delivery before 37 weeks of complete gestation, is a major cause of perinatal mortality and morbidity. Prematurity is also associated with long term complications including developmental delay, and central nervous system disorders [1]. The difference in prevalence of preterm birth observed between self-reported African American and European American race (17.8% and 11.5% respectively) remains largely unexplained and contributes to 3.4 times more African American infant deaths [2]. The challenge of identifying the factors contributing to this difference is hampered by the lack of knowledge about the etiology of preterm birth. It is thought to be heterogeneous and multifactorial involving both genetic and environmental contributions [3], [4], [5], [6].

While salient risk factors for preterm birth have been indentified, ambiguity about the overall contribution of genetic and environmental sources remains. Previous preterm birth associated with an increased odds of 5.9 (95% C.I. = 4.1, 8.6) for a subsequent preterm birth [7], may involve not only fetal and maternal genetic sources that are shared in successive births, but also environmental exposures common to all pregnancies of individual mothers. Similarly, the increased risk associated with self-identified African American race (odds ratio = 1.4 (95% C.I. = 1.1, 1.8), referent = white; reported in Lang, et al. [7]) may be attributed to allelic differences between racial groups [8], [9] or to environmental exposures more prevalent in one group compared with the other, or both.

A further complication in understanding whether the genetic and environmental risks that track with these variables contribute to prematurity *per se,* is how these factors differ between racial groups. Although many environmental risk factors for preterm birth, such as low socioeconomic status [10], increased stressful life events [11], and poorer prenatal care [12], are more commonly observed in African Americans versus European Americans, models of social, psychosocial and economic disparities have failed to account for the racial difference in preterm birth rates to an appreciable extent [10], [11], [13]. Several studies have demonstrated that culturally-defined categories such as race correlate closely with genetic clusters implying that allelic differences may partly explain between group phenotypic differences [14], [15]. Yet, the limited understanding of the contribution of genes to phenotypic variation precludes the testing of assumptions regarding genetic contributions to racial differences [16], [17].

Although there is no obligatory connection between the causes of differences in preterm birth rates *between* racial groups and those responsible for variation *within* groups, knowledge of the latter may inform the former, especially if there are known racial disparities in environmental covariates within groups. Quantitative genetic methods can be used separate phenotypic variation into fetal genetic, maternal genetic, familial environmental and pregnancy-specific environmental components [3]. In this study we show how the pattern of covariances in a large mixed race sample of Virginia siblings, half-siblings and the children of twins provide sufficient information to describe a comprehensive picture of genetic and environmental racial heterogeneity and offers direction for future research.

## Results

The prevalence of gestational ages less than or equal to 37 weeks was 14.7% in European Americans and 20.6% in African Americans. At a threshold of less than 37 weeks the percentages were 7.8% and 11.9% for European American and African Americans respectively. The average adjusted gestational age of African Americans of 38.91 weeks was significantly less than the average value for European Americans of 39.39 weeks (*p*-value <0.001; refer to model 3 in Table 1). The maximum likelihood estimate of variance in gestational age for African Americans was 7.097, almost twice as large as that observed for European Americans (σ^2^ = 3.764). Although the difference in mean values of gestational age between racial groups was expected the difference in variance was not.

**Table 1:** Indices of model fit to assess within-group genetic and environmental contributions and between-group racial heterogeneity.

Model	Description	*-*2LL	*k*	AIC	*p*-value
1	Full model	3076345.0	17	-	-
2	Half-sibling environmental parameter *h* omitted	3076344.6	16	1609698.6	0.527
3	Identical group means	3078984.8	15	1612336.8	<0.001
4	No racial heterogeneity	3102695.5	12	1636041.5	<0.001
5	*f* ^2^ equated across race	3076348.4	15	1609700.4	0.051
6	*m* ^2^ equated across race	3076350.7	15	1609702.7	0.014
7	*c* ^2^ equated across race	3076368.1	15	1609720.1	<0.001
8	*e* ^2^ equated across race	3076477.3	15	1609829.3	<0.001
9	EA *f* ^2^ omitted	3076425.2	15	1609777.2	<0.001
10	EA *m* ^2^ omitted	3076410.6	15	1609762.6	<0.001
11	EA *c* ^2^ omitted	3076362.0	15	1609714.0	<0.001
12	AA *f* ^2^ omitted	3076344.9	15	1609696.9	0.584
13	AA *m* ^2^ omitted	3076373.5	15	1609725.5	<0.001
14	AA *c* ^2^ omitted	3076386.5	15	1609738.5	<0.001

The table presents the following values: −2 times the log likelihood (-2LL), the number of free parameters in the model (*k*), an index of the balance between goodness of model fit and parsimony (Akaike's information criterion, or AIC), and the *p*-value significance result from the likelihood ratio test (distributed as a chi-square statistic). The fit of model 2 was compared to model 1 and all subsequent models were compared to model 2. Significant *p*-values indicate a significant reduction in model fit. AA =  African American, EA =  European American, *f* ^2^ =  fetal effect, *m* ^2^ =  maternal effect and *c* ^2^ =  shared environmental effect.

Table 1 summarizes model-fitting statistics for several models for the source and magnitude of factors contributing to variation in gestational age and their heterogeneity between races. The full model (Model 1 in Table 1) allowed for the effects of fetal genetic (*f* ^2^), maternal genetic (*m* ^2^), shared environment (*c* ^2^) and unique environment (*e* ^2^) to take unique values in each race. This model also included a parameter, *h*, to allow for differences in the contribution of the shared (familial) environment between full and half-siblings. Compared to model 1, a nested model (model 2) with *h* removed resulted in a non-significant degradation in model fit and indicated that this parameter could be omitted. All subsequent nested models (with fewer parameters) were compared to model 2. Models 4 to 8 indicated that the variance components could not be equated across racial groups and provided evidence for both genetic and environmental heterogeneity. The sequential omission of variance components in models 9 to 14 showed that dropping the *f* ^2^ contribution for African Americans provided the most parsimonious fit to the data. In summary, tests indicated the presence of race-specific effects of the fetal genotype, *f* ^2^, the maternal genotype, *m* ^2^, non-genetic effects shared by successive pregnancies of the same mother, *c* ^2^, and random environmental effects specific to individual pregnancies, *e* ^2^. Table 2 shows estimates and confidence intervals of variance components and proportions of variance for the best fitting and full genetic model. Fetal genetic factors explain up to 35.2% of variability of gestational age in European American (EA) and a negligible amount in African American (AA) births. The timing of gestation in AAs was more sensitive to: i) the effects of the maternal genome (*m* ^2^_AA_ = 1.040 versus *m* ^2^_EA_ = 0.503); ii) environment factors shared across siblings (*c* ^2^_AA_ = 1.281 versus *c* ^2^_EA_ = 0.264) and; iii) environmental factors unique to each pregnancy (*e* ^2^_AA_ = 4.777 versus *e* ^2^_EA_ = 1.674).

**Table 2:** Estimated variance components from model 2 with empirically derived 95% bootstrap confidence intervals adjusted for covariates (birth order, maternal age, fetal sex, source of care, smoking, maternal education).

	Full Genetic Model (Model 2)			Reduced Genetic Model (Model 12)		
Source	Estimate	95% CI	Percentage	Estimate	95% CI	Percentage
*African American*						
Fetal genetic	0.264	(0.0, 2.302)	3.7	-	-	-
Maternal genetic	0.976	(0.274, 1.357)	13.8	1.040	(0.531, 1.445)	14.7
Shared environment	1.215	(0.499, 1.666)	17.1	1.281	(0.872, 1.781)	18.0
Unique environment	4.642	(3.559, 4.899)	65.4	4.777	(4.625, 4.927)	67.3
*European American*						
Fetal genetic	1.325	(0.640, 1.927)	35.2	1.325	(0.695, 1.964)	35.2
Maternal genetic	0.503	(0.263, 0.767)	13.4	0.503	(0.235, 0.758)	13.4
Shared environment	0.263	(0.006, 0.537)	7.0	0.264	(0.027, 0.537)	7.0
Unique environment	1.673	(1.355, 2.024)	44.4	1.674	(1.355, 1.990)	44.5

## Discussion

Our results show that the significant racial difference in the variance of gestational age can largely be attributed to non-genetic sources that contribute to differences between successive pregnancies of the same mother and between sibships. Taken together, these contributions were 3.1 times greater in African Americans versus European Americans. For both racial groups the magnitude of unique environmental influences was approximately twice as large as the combined effect of maternal genetic and shared environmental factors, which operate to create stability in the uterine environment within sibships. This suggests that the observed racial difference in variance of gestational age was due in large part to the effect of greater environmental heterogeneity in African Americans. This greater environmental variance generates larger differences among successive births to the same mother, as opposed to stable differences which would affect all pregnancies of the same mother.

Substantial heterogeneity in the effect of environmental exposures were detected between racial groups even after accounting for differences that can be ascribed to fetal and maternal genetic influences. These results also persist over and above the effects of multiple covariates known to correlate with prematurity. With the exception of maternal education, which changes to a small degree over successive pregnancies, the remaining covariates were pregnancy-specific and are expected to diminish the large effect we observe for the unique environment. The non-overlapping 95% confidence intervals (Table 2) for both environmental parameters along with the highly significant deterioration in model fit (models 7 and 8, Table 1) suggests that a large remainder of race-specific environmental variance is not accounted for by these covariates. Omitting the covariates from the model had a negligible effect on the race differences in means, variances and genetic and environmental parameter estimates.

We corroborated the contribution of fetal and maternal genetic factors to variation in gestational age [3], [18], [19] and showed in our study fetal genetic effects were exclusively present in the European American sample. The null contribution of fetal genetic factors to variation in African American gestational age may reflect the consequences of a large contribution of unique environmental sources. Estimates of these components are negatively correlated because they both contribute to estimates of differences between individual pregnancies of the same mother. Attempts to equate either the fetal or maternal genetic contribution across races resulted in significant degradation in model fit statistics and suggests a differential contribution of fetal and maternal genes. Yet differences in genetic contributions between races were modest even in this very large sample compared to the large differences reported for environmental factors. This gives considerable weight to further identifying environmental exposures that contribute to the increase heterogeneity observed in African Americans. We note that the differences in point estimates of genetic parameters could reflect either true differences in genetic variance between races or gene by environment interaction (GxE).

The rate of births before 37 weeks gestation has increased by 21% from the period of 1989 to 2006 with consistent differences observed between races over this period [12]. Yet, despite the clear documentation of this public health problem and racial disparity, little progress has been made in identifying the antecedents of preterm birth. The results of this study suggest four avenues for further research. First, the largest contribution to differences in gestational age both within and between groups was pregnancy-specific environmental sources. Future studies could profitably focus on identification of exposures that change between pregnancies and characterize the observed increased reproductive heterogeneity in African American mothers. Second, the environment common over all pregnancies of the same mother has a sizable effect in explaining between family differences between African Americans versus European Americans. Although the overall effect of the shared environment was smaller than that of unique environmental sources, its significant effect suggests that there is a pervasive contribution of familial characteristics which may include social and economic factors. Third, interaction between these two environmental sources may be an additional source of increased gestational age heterogeneity in African Americans. For example, access to prenatal health care may not only be less available to African Americans due to higher poverty levels but also, when available, unpredictably so over successive pregnancies. Fourth, in the present study the contribution of fetal and maternal genetic factors was significant but explained less variability in gestational age both within and between racial groups than either environmental source. Further modeling of genetic effects could incorporate possible interactions between genetic variation and sources of the large difference in environmental heterogeneity observed between races. For instance, the increased preterm birth risk associated with bacterial vaginosis, which is more prevalent in African Americans, is modified by a rare variant in the promoter region of TNF [20]. Examination of the genetic sensitivity to environments would not be restricted to loci that differ in allele frequencies between racial groups. Overall, these additional research directions are consistent with both investigation of exposures during pregnancy and models of exposures over the life-course that influence reproductive potential [21].

In summary, we report quantitative genetic analyses in a large sample of Virginia families to describe how genetic and environmental factors contribute to differences in variability of gestational age between Americans of European and African ancestry. Environmental factors, particularly environmental exposures that differ across pregnancies, were largely responsible for the increased variability in the timing of African American births compared with European Americans. This greater environmental variation of African American births could be, for instance, a reflection of the greater unpredictability in accessing prenatal care or to their greater vulnerability to the effects of random non-genetic influences via genetic and/or social mechanisms. Future genetic studies may best be directed to understanding the racial differences in the socio-cultural sources of this heterogeneity, and their possible interaction with genetic differences within and between races. Otherwise, in order to further our understanding of the observed racial disparity in preterm birth, these results argue for greater resources to be invested in the identification and measurement of environmental influences that are less stable over successive pregnancies in African Americans versus European Americans.

## Methods

### Study Sample

Pregnancy histories were obtained by combining the results of two separate requests for birth records from the Virginia Department of Health Office of Vital Records. A data-merge identified full and half-sibships by combining birth records that shared parental social security numbers (SSN) from Virginia births between 1989 and 2008. Individuals in full-sibships were required to share both the maternal and paternal SSN, while individuals in maternal half-sibships shared only the maternal SSN and those sharing only the paternal SSN were identified as paternal half-sibships. Records with either parental SSN missing were excluded. The result of this match was combined with a second set of birth records obtained from a previous study [3] comprising the offspring of twin parents identified through the Mid-Atlantic Twin Registry [22]. Records were obtained by matching the SSNs of registered twins against parental SSNs on birth records held by the Office of Vital Records. The Virginia Commonwealth University IRB approved the study design, sample collection and waiver of informed consent (VCU IRB# HM11443). Informed consent was not required since personally identifiable information was not sent to the authors from either the MATR or VDH. Birth outcome exclusion criteria included multiple birth, any congenital anomalies, hydramnios/oligohydramnios, pregnancies complicated by pregnancy induced hypertension and eclampsia, Rh sensitization, abruptio placenta and placenta previa, or any medically necessitated preterm delivery. Gestational age was recorded as completed weeks as estimated by the physician. For each birth record race was classified as African American if the child's race and the race of both parents was listed as non-Hispanic Black and European American if the child's race and the race of both parents was listed as non-Hispanic White. After screening, the sample used in this study consisted of 733,339 births of which 17.8% were classified as African American (Table 3).

**Table 3:** Sample frequencies by parental relationship and race.

	European American		African American	
Parental relationship	N. Families	N. Births	N. Families	N. Births
Sibship	284,446	575,709	66,983	119,791
Maternal half-sibship	6,736	12,269	2,431	4,515
Paternal half-sibship	5,419	9,800	2,839	5,292
MZ male twin	595	1,092	69	99
MZ female twin	618	1,212	98	144
DZ male twin	393	700	52	77
DZ female twin	368	696	72	119
DZ male-female twin	936	1,614	139	210
*Total*	299,511	603,092	72,683	130,247

### Model for Maternal and Fetal Effects

Expectations for genetic and environmental contributions to variances and covariances of relatives are derived from biometrical genetic theory [23], [24], [25]. The decomposition of within group phenotypic variation for birth outcomes can be described as a weighted combination of fetal (*f* ^2^) and maternal (*m* ^2^) genetic and shared (*c* ^2^) and unique (*e* ^2^) environmental latent variables. The proportion of genetic influences shared between related individuals (biologically or otherwise) is inferred by the laws of segregation assuming random mating. By this model, the covariance between sibling births can be explained by the one-half of genes they share (½*f* ^2^), the maternal genetic factors from a common mother (*m* ^2^) and aspects of the familial environment that they share (*c* ^2^). Unique environmental factors (*e* ^2^) are not shared and account for pregnancy specific environmental exposures in addition to measurement error. The covariance between maternal half-siblings would differ in that they share one-fourth of their genes in common (¼*f* ^2^+*m* ^2^+*c* ^2^). Using these expectations a rough estimate of fetal genetic influences (*f* ^2^) on phenotypic variance can be derived by subtracting four times the difference of the full-sibling and maternal half-sibling correlation (*f* ^2^ = 4((½*f* ^2^+*m* ^2^+*c* ^2^) − (¼*f* ^2^+*m* ^2^+*c* ^2^))).

All individuals who share maternal genetic influences also share the contribution of fetal genes, yet the converse is not true if one considers paternal half-siblings. Thus, an estimate of maternal genetic influences (*m* ^2^) can be derived by subtracting the correlation of paternal half-siblings from maternal half-siblings since both relationships share one-fourth of their fetal genes but only maternal half-siblings share mothers in common (*m* ^2^ = (¼*f* ^2^+*m* ^2^+*c* ^2^) − (¼*f* ^2^+*c* ^2^)).

Additional relationships beyond full and half-sibships need to be considered to distinguish the influence of shared and unique environmental from genetic sources (Table 4). The offspring of monozygotic (MZ) twins, like other biological half-siblings, share one-fourth of their genes (*f* ^2^) in common, while the offspring of dizygotic (DZ) twins, like other first cousins, share one-eighth their genetic load. Cousins related through MZ female twins would also share all of their maternal genetic influence but not the effects of the shared environment since they are members of different sibships. Accordingly, an estimate of the shared environment can be calculated by subtracting the correlation of the offspring of MZ female twins from the maternal half-sibship correlation (*c* ^2^ = (¼*f* ^2^+*m* ^2^+*c* ^2^) − (¼*f* ^2^+*m* ^2^)). An estimate of unique environmental sources can be obtained by subtracting both the genetic and common environment from the total phenotypic variance (*vt* ^2^), *e* ^2^ = *vt* ^2^ *− f* ^2^ *− m* ^2^ *− c* ^2^. The importance of a factor accounting for within group variance can be calculated as the proportion of variance explained relative to total variance; thus the proportion of fetal genetic variance is calculated as *f* ^2^/(*f* ^2^ + *m* ^2^ + *c* ^2^ + *e* ^2^).

**Table 4:** Expected covariance of gestational age expressed as variance components between pregnancy outcomes as a function of relationship between offspring.

Parental relationship	Familial relationship of offspring	Expected covariance
MZ female twins	Cousin	¼ *f* ^2^ + *m* ^2^
DZ female twins	Cousin	⅛ *f* ^2^ + ½ *m* ^2^
MZ male twins	Cousin	¼ *f* ^2^
DZ male twins	Cousin	⅛ *f* ^2^
DZ male-female twins	Cousin	⅛ *f* ^2^
Full sibship	Sibling	½ *f* ^2^ + *m* ^2^ + *c* ^2^
Maternal half-sibship	Half-sibling	¼ *f* ^2^ + *m* ^2^ + *hc* ^2^
Paternal half-sibship	Half-sibling	¼ *f* ^2^ + *hc* ^2^

*f* ^2^ = fetal genetic,

*m* ^2^ = maternal genetic,

*c* ^2^ = shared familial environment.

*h* = parameter to allow for differences in half-sibling versus full-sibling shared environment.

Although it is instructive to derive estimates of genetic and environmental effects using correlations between relatives, in practice, structural equation modeling is preferred to make a simultaneous decomposition of the covariance matrix using widely available software implementing maximum likelihood [26], [27], [28] or Bayesian [29], [30] approaches. These methods yield confidence intervals of parameter estimates and goodness-of-fit indices quantifying how well the model accounts for the empirical variances and covariances and enabling the testing of hypotheses regarding the causes of variation within groups and their heterogeneity between groups.

### Parameter Estimation and Hypothesis-Testing

A convenient feature of structural equation methods is the ease in which families composed of different relationships and sizes can be incorporated [31]. Expectations for covariance matrices were specified for each sibship and children of twins family type based on the equations in Table 4. We followed the model specification as described in York *et al*. [3] for continuous outcomes in which multiple births are treated as repeated measures within the same family. In contrast to methods that pool births from the same mother, this treatment maintains the information content of each family and allows for the inclusion of measured covariates that may differ across births. Model assumptions included: (1) random mating; (2) genetic effects were additive and constant over pregnancies; (3) the influence of fetal and maternal genetic differences are the same for male and female fetuses (*i.e.*, genetic effects are autosomal and neither X-linked nor sex-limited); (4) genetic and environmental variables do not interact and; (5) environmental effects were pregnancy specific apart from the effects of maternal genotype, shared environmental effects, measured covariates and other aspects of the parental phenotype (*e.g.*, cultural inheritance).

To balance computation time with gains in information sibships were limited to the first four reported births, which corresponded to 96.7% of available births. Measured covariates were included based on prior evidence of association with preterm birth risk or mean levels differed between race, namely: birth order, maternal age, maternal education, source of care (private physician or other), fetal sex and number of reported cigarettes smoked daily while pregnant. Maximum likelihood estimates of the means and expected covariance matrices were obtained using the structural equation modeling program Mx [26]. A test of heterogeneity was performed by equating the genetic and environmental parameters across racial groups and assessing the decline in model fit. The contribution of individual parameters were examined by dropping each in turn from the model and observing the decline in fit of the submodel by the likelihood ratio chi-square test and change in the Akaike Information Criterion (AIC) in an attempt to arrive at a model yielding the optimal balance of parsimony and goodness-of-fit. Confidence intervals for the genetic and environmental parameters were obtained from 1,000 iteration bootstrap estimates by randomly sampling the families with replacement to generate samples with the same number of families.
